# X-ray scatter in projection radiography

**DOI:** 10.1093/rpd/ncad275

**Published:** 2023-11-03

**Authors:** Satu Ylimaula, Lasse Räsänen, Miia Hurskainen, Arttu Peuna, Petro Julkunen, Miika Tapio Nieminen, Matti Hanni

**Affiliations:** Research Unit of Health Sciences and Technology, Faculty of Medicine, University of Oulu, 90220 Oulu, Finland; Department of Diagnostic Radiology, Oulu University Hospital, 90220 Oulu, Finland; Department of Diagnostic Radiology, Oulu University Hospital, 90220 Oulu, Finland; Terveystalo Healthcare, 00100 Helsinki, Finland; Department of Technical Physics, University of Eastern Finland—Kuopio Campus, 70210 Kuopio, Finland; Department of Diagnostic Services, Hospital Nova of Central Finland, Wellbeing Services County of Central Finland, Hoitajantie 3, 40620 Jyväskylä, Finland; Department of Technical Physics, University of Eastern Finland—Kuopio Campus, 70210 Kuopio, Finland; Department of Clinical Neurophysiology, Kuopio University Hospital, 70200 Kuopio, Finland; Research Unit of Health Sciences and Technology, Faculty of Medicine, University of Oulu, 90220 Oulu, Finland; Department of Diagnostic Radiology, Oulu University Hospital, 90220 Oulu, Finland; Medical Research Center Oulu, University of Oulu and Oulu University Hospital, Oulu, Finland; Research Unit of Health Sciences and Technology, Faculty of Medicine, University of Oulu, 90220 Oulu, Finland; Department of Diagnostic Radiology, Oulu University Hospital, 90220 Oulu, Finland; Medical Research Center Oulu, University of Oulu and Oulu University Hospital, Oulu, Finland

## Abstract

Projection radiography is the most common radiological modality, and radiation safety of it concerns both radiation workers and the public. We measured and generated a series of scattered radiation maps for projection radiography and estimated effective doses of the supporting person during exposure. Measured adult patient protocols included chest posterior–anterior, chest lateral, pelvis anterior–posterior (AP), abdomen AP and bedside chest AP. Maps concretise spatial distribution and the scattered radiation dose rates in different imaging protocols. Highest and lowest rates were measured in abdomen AP and bedside chest AP protocols, respectively. The effective dose of supporting person in abdomen AP examination at distance of 0.5 m was 300 nSv and in bedside supine chest AP examination at distance of 0.7 m was 0.5 nSv. The estimated annual effective dose of emergency unit radiographer was 0.11 mSv. The obtained effective dose values are small compared to annual dose limits of radiation workers and the public.

## Introduction

Healthcare professionals can be exposed to ionising radiation in different X-ray based imaging modalities. Occupational exposure is mainly caused by scattered radiation, which is generated by any object on the path of the X-ray primary beam^(1)^, the principal source being the patient. The dose received by the personnel in a single examination is small compared to the patient dose, but the cumulative dose during a long work career can be significant^(2)^. This kind of frequently occurring low-dose radiation exposure can be connected with a small excess risk of cancer^(3–5)^. Even though the excess risk for individual is small, the overall stochastic effects for population can be considerable^(6)^.

In addition to the imaged patient and the healthcare personnel there can be other patients or family members in the room during imaging. For instance, when mobile radiography is used it is not uncommon to have other patients in the room during imaging. Also, it is not uncommon for a patient to need support during imaging, and in these situations either family or staff member stays in the imaging room during exposure.

The radiation dose of the patients, healthcare professionals and supporting persons can be minimised by following radiation protection guidelines. Proper training is an essential part of these. In Finland, the arrangement of radiation protection training for the radiation workers is part of radiation law^(7)^. Based on the EU directive 2013/59/EURATOM, similar training practices are required in all EU countries^(8)^.

The knowledge of how to minimise exposure within the imaging room is an important part of radiation safety training. Visualisation of scattered radiation is an effective tool for this purpose. This can be done either based on simulations or measurements using dosemeters^(9–15)^. Most of the recently published studies have focused on the interventional radiology procedures, since personnel conducting them are amongst the highest exposed healthcare workers^(16)^. However, projection radiography is the most common imaging modality used in radiology^(17, 18)^, and the matter of radiation safety in it raises questions amongst personnel and the general public^(19, 20)^.

In this study, we measured scattered radiation at specific locations in the imaging rooms and generated a series of scattered radiation maps for projection radiography. These maps are intended to be used in the radiation protection training of healthcare personnel. Additionally, maps offer a way to estimate the exposure of the supporting person or other patients in the room and may help to minimise the resulting exposure by providing a tool to optimise positions of personnel within the room. Different imaging protocols were used in the measurements to identify the spatial distribution of the scattered radiation in various situations. The included adult patient protocols were chest posterior–anterior (PA), chest lateral (LAT), pelvis anterior–posterior (AP), abdomen AP and bedside chest AP radiographic projections. Chest protocols were selected due to their popularity^(21)^, pelvis and abdomen protocols due to the high amount of scatter expected in them and bedside chest protocol due to commonly encountered exposure of personnel and other patients occurring in it. Generated maps visualise the well-known inverse square law of radiation intensity and reveal possible areas of low-dose rate scattered radiation, as well as differences on the amount of scatter between protocols. An additional purpose of this study was to estimate the effective dose of the assisting person and other patients within the imaging room during exposure.

## Methods

In this study, an ATOM dosimetry verification phantom 701/C (Sun Nuclear, USA) was used as a scattering object. It is an anthropomorphic adult male phantom, which comprises of 25-mm-thick sections and includes tissue equivalent structures. An active-type dosemeter system consisting of semiconductor detectors (Raysafe i2, Unfors Raysafe AB, Sweden) was used to collect the dose rate values of the scattered X-ray radiation. Dosemeters were calibrated in the appropriate manner by the manufacturer. The dosemeter system monitors the personal dose values of the healthcare personnel using an operational measurement quantity H_p_(10), which is the personal dose equivalent at tissue depth of 10 mm.

**Figure 1:**
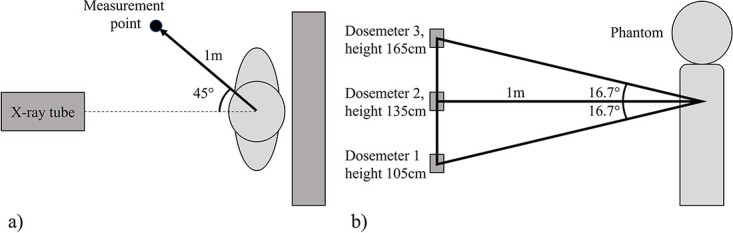
Illustration of representative dosemeter positions in the measurements with respect to the centre point of the scatter origin inside phantom: (**a**) top view and (**b**) side view. The actual heights, angles and distances varied between measurements (Table 1). Presented positions used in one chest PA/LAT measurement and in all repeatability measurements

Three dosemeters were attached to an infusion stand at three different heights (Figure 1)^(22)^. Installation heights varied between measurement setups. The centremost dosemeter was positioned close to the height, where radiation beam enters the phantom. The other two dosemeters were positioned vertically ±35 cm or ±30 cm from the centremost dosemeter depending on the measurement setup. Installation heights of the uppermost and the lowest dosemeters were selected rather close to the centremost due to known angular dependence of the dosemeter^(23)^.

**Figure 2:**
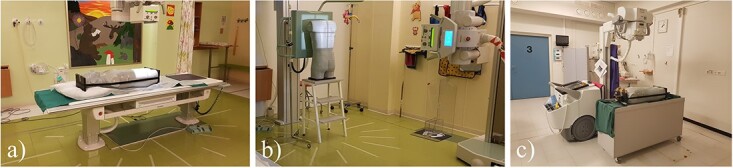
Measuring setups used for (**a**) supine pelvis and abdomen AP, (**b**) standing chest PA and (**c**) supine chest AP

**Figure 3:**
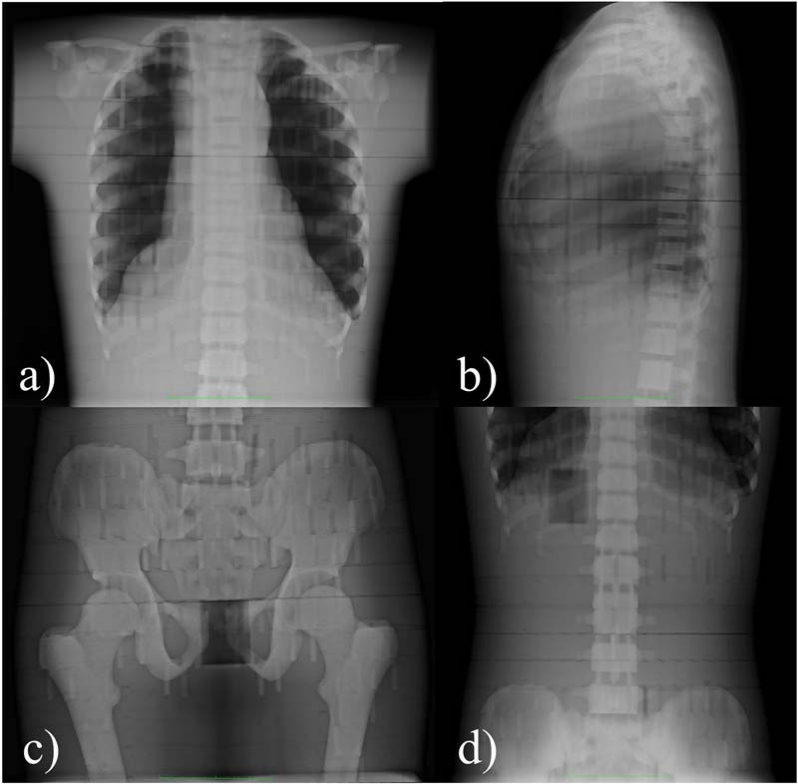
Imaged area in (**a**) chest PA, (**b**) chest LAT, (**c**) pelvis AP and (**d**) abdomen AP setups

Measurements were carried out in two X-ray examination rooms at Oulu University Hospital. In the first room, a stationary radiography equipment of the room (Fujifilm FRD AcSelerate, Japan) was used to acquire standing chest PA and LAT radiographs, supine pelvis AP radiographs and abdomen AP radiographs. In the second room, a mobile radiography device (Philips MobileDiagnost wDR, the Netherlands) was used to acquire supine chest AP radiographs. Figures 2 and 3 present images of the measuring setups and the obtained radiographs, and Table 1 includes details of each setup.

**Table 1:** Imaging-related parameters: imaging protocol, imaging device, posture, dosemeter installation heights, measurement distances, tube voltage and tube current time product. In chest examinations, grid specifications were 67.6 lines/cm, ratio 14/1 and FFD 180 cm and in pelvis/abdomen examinations, 67.6 lines/cm, ratio 10/1 and FFD 120 cm.

Imaging protocol	Imaging device, total filtration	Posture	Dosemeter installation heights (cm)	Measurement distances (m)	Tube voltage (kVp), tube current time product (mAs)
Chest PA	Fujifilm FDR AcSelerate with grid, 0.2 mm Cu + 2.7 mm Al	Standing	105, 135, 165	0.5, 1, 1.5, 2, 2.5, 3	125, 1.75
Chest PA with pelvic shield					
Chest LAT					125, 8.5
Pelvis AP	Fujifilm FDR AcSelerate with grid, 0.1 mm Cu + 2.7 mm Al	Supine	50, 80, 110		81, 16.5
Abdomen AP					82, 15
Thorax AP	Philips MobileDiagnost wDR without grid, 0.2 mm Cu + 4.3 mm Al		50, 85, 120	0.7, 1, 1.3, 1.6, 1.9	125, 1.7

**Figure 4:**
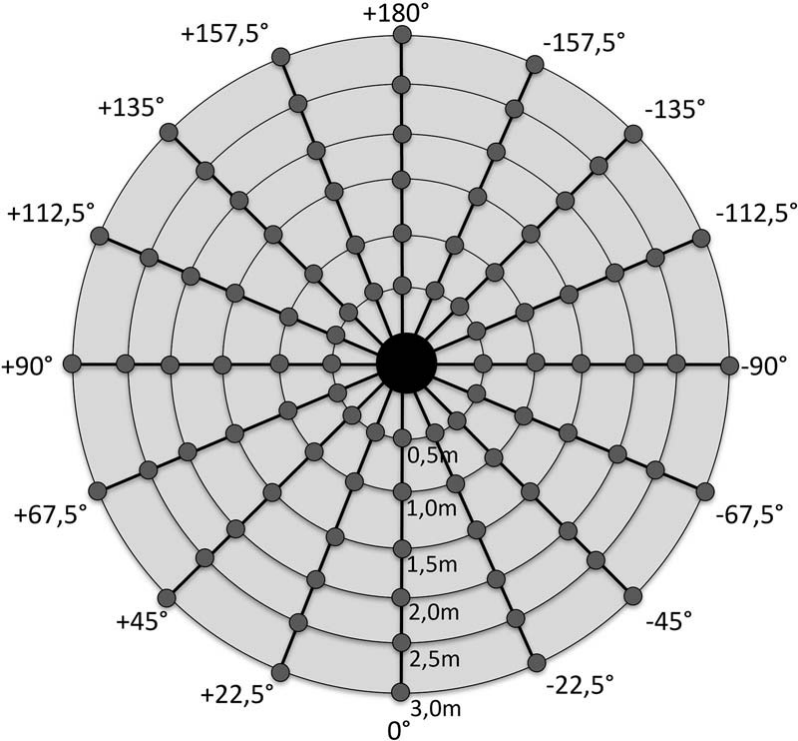
Schematic figure of the used measurement points (small grey dots) with respect to the scattering object (big black dot in the centre). With the mobile imaging device, measurement distances were 0.7, 1, 1.3, 1.6 and 1.9 m

Measurements were performed circularly around the scattering object. The angular difference between adjacent measurement points was 22.5° (Figure 4). Measurement distances were 0.5, 1, 1.5, 2, 2.5 and 3 m from the scatter origin, except with the mobile imaging device, with which the distances were 0.7, 1, 1.3, 1.6 and 1.9 m due to expected low scatter values and restricted space. In some parts of the room fixed structures, imaging equipment and dosemeter holder prevented the measurements. In addition, in standing chest radiography the primary beam area was excluded from the measurements, as the manufacturer does not recommend using dosemeters in the primary beam.

The MATLAB programme^(24)^ (MATLAB22b, MathWorks, USA) was used to generate scattered radiation maps based on the measured dose rate values. In addition, these dose rate values were used to estimate the effective dose of supporting persons and other patients staying in the imaging room. Personal dose equivalent values, H_p_(10), can be used in the assessment of the effective dose under the assumption of a uniform whole body exposure. To calculate the effective dose, the measured H_p_(10) dose rate values were multiplied with the exposure times, extracted from the DICOM metadata.

Repeatability of the chosen measurement method was evaluated by repeating dose rate measurement 10 times at a representative point at three heights at the distance of 1 m from the phantom. Consecutive measurements were carried out at intervals of 2–5 min and the dosemeter holder was repositioned after each measurement. Figure 1 illustrates positions of the measurement points used in repeated measurements with respect to the centre of the scatter origin inside the phantom, i.e. with respect to the primary beam field centre point inside phantom. The highest measurement accuracy was achieved with the dosemeter that was positioned on the same horizontal plane with the scatter origin (Table 2). The achieved standard deviation and coefficient of variation were the lowest with this dosemeter.

**Table 2:** Measurement accuracy achieved with dosemeters at different heights at 10 repeated measurements.

	Dosemeter 1, 105 cm	Dosemeter 2, 135 cm	Dosemeter 3, 165 cm
Mean (μSv/h)	1100	1020	950
Standard deviation (μSv/h)	180	50	190
Coefficient of variation (%)	16	5	20

## Results

### Scattered radiation maps

**Figure 5:**
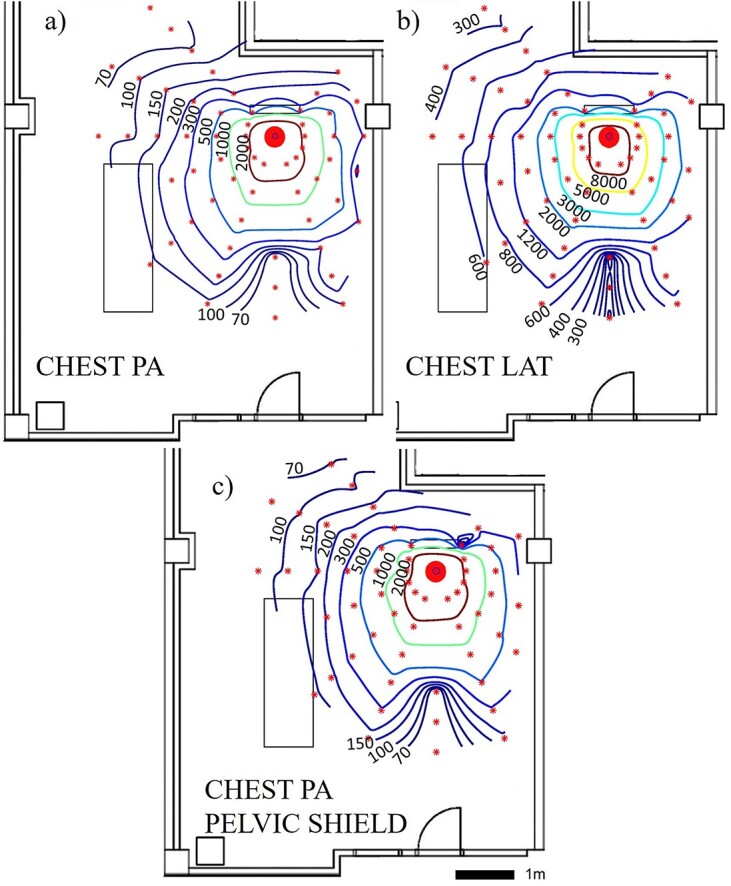
Scattered radiation maps acquired in standing (**a**) chest PA, (**b**) chest LAT and (**c**) chest PA with pelvic shield setups. Scattered radiation on the isodose lines is presented in microsieverts per hour at the height of 135 cm. The large dot indicates the position of the imaged phantom and smaller star-shaped dots the measurement points

In chest radiography, the scatter in LAT projection was larger than scatter in PA projection (Figure 5a and b). At 1-m distance, the average measured dose rate in LAT projection was over 300% larger compared to the PA projection (Table 3). The use of pelvic shield affected the maps only negligibly (Figure 5c), as the average dose rate measured with pelvic shield at 1-m distance was only 12% higher compared to the same measurements without shielding (Table 3). The scatter was strongly backscattered towards the X-ray tube when the horizontal plane is considered (Figure 5a–c). Differences in the amount of scatter at different measurement heights were small compared to the achieved coefficient of variation (Table 2). Thus, only the maps acquired at the height of 135 cm are presented in Figure 5. The average difference of dose rates of uppermost and lowermost maps compared to the centremost map were −22 and 1% in PA projection and −17 and 10% in LAT projection, respectively.

**Table 3:** Average measured dose rates of different examinations at a distance of 1 m and differences (%) compared to the standing chest PA examination values with the same distance and at 135 cm height.

Examination, height of measurement	Average dose rate at 1 m distance (μSv/h)	Average dose rate compared to the chest PA examination (%)
Supine abdomen AP, 80 cm	7300	+958
Supine pelvis AP, 80 cm	3940	+471
Standing chest PA, 135 cm	690	—
Standing chest LAT, 135 cm	2790	+304
Standing chest PA pelvic shield, 135 cm	770	+12
Supine chest AP, 85 cm	250	−64

**Figure 6:**
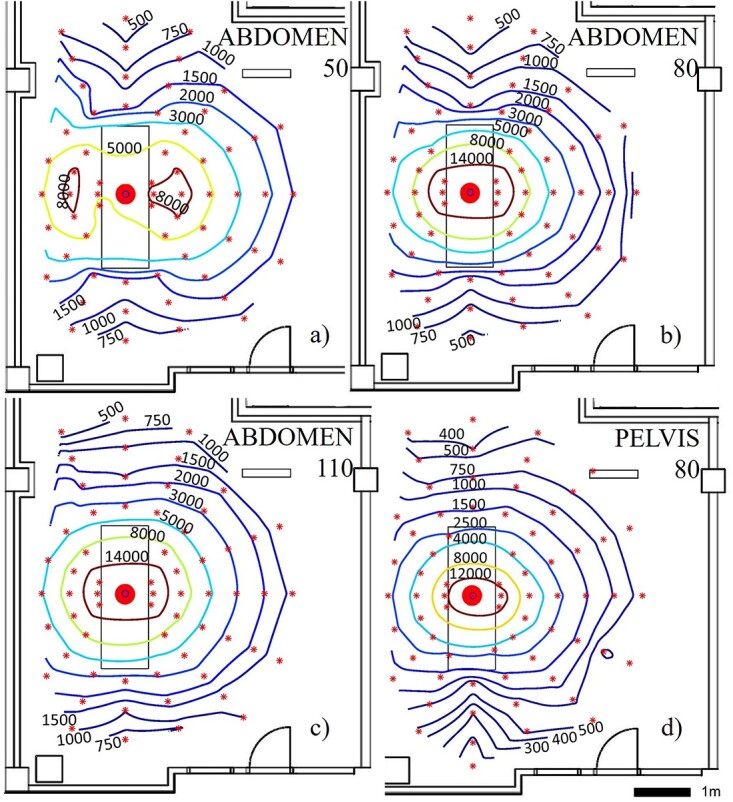
Scattered radiation maps acquired in supine abdomen AP protocol at the heights of (**a**) 50 cm, (**b**) 80 cm and (**c**) 110 cm and supine pelvis AP protocol at the height of (**d**) 80 cm. Scattered radiation on the isodose lines is presented in microsieverts per hour. The large dot indicates the position of the imaged phantom and smaller star-shaped dots the measurement points

In supine pelvis AP and abdomen AP (Figure 6) protocols, the amount of scatter was the largest of all studied protocols. Average dose rates in these examinations at 1-m distance and at the height of 80 cm were over 400 and 900% larger compared to the chest PA examination values at 135-cm height and at the same distance (Table 3), respectively. Scatter was oriented nearly isotropically to all directions at the plane perpendicular to the X-ray primary beam. The scattered radiation map at the height of 50 cm (below bucky table) had an asymmetrical shape (Figure 6a).

**Figure 7:**
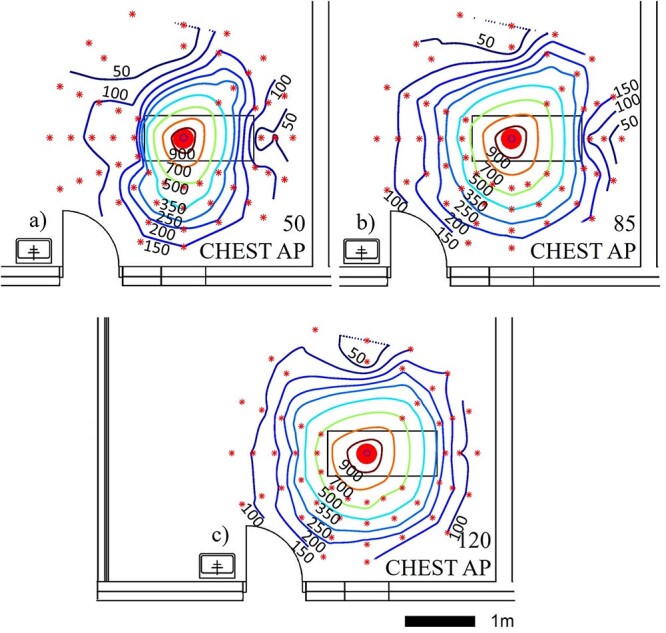
Scattered radiation maps acquired with mobile imaging device using supine chest AP imaging protocol at the heights of (**a**) 50 cm, (**b**) 85 cm and (**c**) 120 cm. Scattered radiation on the isodose lines is presented in microsieverts per hour. The large dot indicates the position of the imaged phantom and smaller star-shaped dots the measurement points

In supine chest AP mobile radiography (Figure 7) the scatter was lowest of all included protocols, and the scatter was oriented nearly isotropically to all directions at the plane perpendicular to the primary X-ray beam. The average measured dose rate in this examination at 1-m distance and at the height of 85 cm was 64% lower than in standing chest PA examination at same distance and at height of 135 cm (Table 3). The scattered radiation map at the height of 50 cm (below patient table level) had an asymmetrical shape (Figure 7a).

### Effective dose values of supporting persons and nearby patients

If a patient needs support in standing chest imaging, an assisting person would most likely be positioned on the side of the patient, roughly 0.5 m away from patient. Measured scattered radiation dose rates at this position were 1700 and 6100 μSv/h (Table 4) in PA and LAT projections, respectively. Furthermore, in supine pelvis or abdomen AP radiography the assisting person could be positioned at the end or at the side of the bucky table. In these situations, the assisting person would be exposed to scattered radiation at dose rates 1350, 14 700, 1250 and 22 200 μSv/h in pelvis and abdomen projections, respectively (Table 4). Effective doses were estimated based on the exposure times obtained from DICOM metadata (Table 4). The highest effective dose was calculated in abdomen AP examination (0.3 μSv), in which also the highest values of scattered radiation at 1-m distance were measured (Table 3).

**Table 4:** Effective dose estimates of assisting personnel and other patients during standing chest PA, chest LAT, supine pelvis AP, abdomen AP and chest AP. Maximum measured dose rates are presented at the specified distances and heights. Also, general exposure times of each protocol during measurements are presented.

Experiment	Distance/height (cm)	Maximum measured dose rate, H_p_(10) (μSv/h)	Exposure time (ms)	Effective dose (μSv)
Standing chest PA	50/135	~1700	11	~0.005
Standing chest LAT	50/135	~6100	28	~0.05
Supine pelvis AP	50/80	~14 700	45	~0.18
	150/80	~1350		~0.02
Supine abdomen AP	50/80	~22 200	50	~0.3
	150/80	~1250		~0.02
Supine chest AP	70/135	~600	3	~0.0005
	160/135	~200		~0.0002

In mobile radiography, we estimate that the radiographer is positioned at a distance of 0.7 m and other patient at a distance of 1.6 m from the imaged patient. In these situations, one is exposed to a scattered radiation at dose rates of 600 and 200 μSv/h, respectively. Estimated effective doses calculated at these distances were on magnitude of nanosieverts (Table 4).

At our hospital the most frequently used conventional radiography device was used to perform 25 000 examinations during 2022. This device is used in three shifts in the hospital’s emergency imaging unit. Most radiography examinations with this and with all other plain radiography devices as well are focused on the chest area. We assume that a fictitious radiographer would work 1 year using this device. We also hypothesize that there would be situations where a patient would need support during imaging and our fictitious radiographer would always act as a supporting person. Additionally, number of examinations occuring annually during this radiographer's shift was assumed to be one-third of the device's examinations, hence 8300. If the amount of support situations would be 1, 10 or 20% of all the examinations, an estimate of the effective dose of the radiographer would be 0.011, 0.11 and 0.22 mSv, respectively. These estimates are based on the conservative assumption that the effective dose value of supporting person in one support situation would be the average of the highest effective doses calculated for standing chest, supine pelvis and supine abdomen (Table 4), hence 0.134 μSv.

## Discussion

In this study, we investigated the spatial distribution of the scattered radiation in the imaging room during an adult’s chest PA, chest LAT, pelvis AP, abdomen AP and bedside chest AP radiography. Based on the measurements scattered radiation maps were generated. Additionally, we estimated the effective doses of the supporting person that remain on the room during imaging. Similar measurement methods were used in all setups to enable fair comparison between protocols.

Even though the estimated scattered radiation doses of assisting persons are low in projection radiography, the popularity of this modality emphasises the importance of them. Many of the previous studies suggest that the excess risk of stochastic effects, such as cancer, is related to the amount of exposure also with small doses^(6, 25)^. Hence, the scattered radiation doses of projection radiography might increase an individual’s risk of cancer slightly. Even if the risk for the individual might be negligible, on the population level this risk can be significant.

The overall shape of acquired scattered radiation in maps is in line with literature in standing chest PA and LAT protocols^(1, 26)^ and in supine AP pelvis, abdomen and bedside chest protocols ^(12, 13, 27)^. However, the metallic structures of patient table attenuate radiation non-uniformly at different distances, which is most likely the reason for the altered scatter maps measured at the lowest height in the supine setups (Figures 6 and 7).

The amount of scatter was largest in the supine abdomen AP imaging protocol (mean dose rate 7300 μSv/h with the centremost dosemeter at 1-m distance) and lowest in the supine chest AP imaging protocol (mean dose rate 250 μSv/h with the centremost dosemeter at 1-m distance). The largest scatter in abdomen AP is expected, as both the image area and the current time product (mAs) were amongst the largest in this protocol compared to other protocols. The measured values of supine chest AP setup are similar magnitude to the values obtained by Abrantes *et al.*^(26)^ and supine abdomen AP values to those obtained by Vlachos *et al*.^(12)^. The patient shield used in the chest PA protocol affected only negligibly to the amount of scattered radiation with the used measurement method when achieved measurement accuracy in repeatability tests is considered. This is expected, as the shield was at the surface of the phantom, outside the primary beam.

Based on the measurements, the lowest effective dose estimate of supporting person was calculated for bedside chest AP measurements (0.0005 μSv at the height of the phantom at 0.7-m distance). The highest effective dose values were calculated for abdomen AP at the height of the phantom on the side of the table (~0.3 μSv at 0.5-m distance). Based on the protocol-specific effective doses, also the annual effective dose value of fictitious emergency unit radiographer was estimated (0.11 mSv with 10% of patients needing support at 0.5-m distance). All of these values are negligible compared to the annual dose limit of the radiation workers (20 mSv)^(7, 28)^. In Finland and in Europe, radiation workers whose annual effective dose can potentially be over 6 mSv are classified as group A radiation workers^(28, 29)^. Based on the results presented here for the fictitious radiographer, there is no need to classify radiographers as group A radiation workers in the studied imaging protocols. When compared to the local background radiation level of the Oulu city district Raksila, ~0.140 μSv/h^(30)^, the mentioned bedside chest AP and abdomen AP effective doses are low as well, as they represent ~12 s and 132 min of the background radiation, respectively. The estimated annual effective dose of a fictitious radiographer (0.11 mSv) represents 33 days of local background radiation. Hence, even though annual dose limits set by regulatory bodies are not exceeded, to diminish possible stochastic effects of radiation on the population level all practical measures should be taken to minimise exposure in the studied radiographic examinations.

When mobile radiography examinations are carried out in the ward, there might be other patients in the room as well. To estimate the effective dose for other patients, we used 1.6-m distance between the patients. The effective dose value at this distance in bedside chest imaging was ~0.0002 μSv, which is equal to ~4 s of local background radiation. Consequently, the effective dose received by other patients is negligible when compared to the annual public exposure limit (1 mSv)^(28)^ and background radiation^(30)^. Thus, it is safe to stay in the premises during imaging without additional shielding.

Dosemeters were attached to the infusion stand during measurements. Thus, the uppermost and lowest dosemeters were not positioned towards the scatter origin inside the phantom. This limits the reliability of the results obtained with these dosemeters due to the angular dependency of the dosemeters^(23)^. Thus, the results obtained with the centremost dosemeter can be considered most reliable. It was positioned in all measurements at the height of the scatter origin, and the angle of scattered radiation reaching it was on average lowest. This assumption is supported by the lowest standard deviation and the lowest coefficient of variation obtained in the repeatability tests. In addition, we employed a phantom without limbs, which may affect the results slightly. If the limbs would be in the imaged area, they would increase the scatter, and if they would be outside the imaged area, they would attenuate the scattered radiation. However, we believe that these effects are minor and do not change the overall conclusions of the study.

## Conclusions

Scattered radiation maps concretise spatial distribution of scattered radiation and reveal differences on the amount of scattered radiation between different imaging protocols. Based on the maps, effective doses were estimated for supporting persons and other patients staying in the room during imaging. In conclusion, the calculated doses are small compared to annual dose limits of both personnel and public set by European regulatory bodies of radiation protection.

## Data Availability

The data presented in this study are available on request from the corresponding author.
