# Correction to: X-ray scatter in projection radiography

**DOI:** 10.1093/rpd/ncad308

**Published:** 2023-12-04

This is a correction to: Satu Ylimaula, Lasse Räsänen, Miia Hurskainen, Arttu Peuna, Petro Julkunen, Miika Tapio Nieminen, Matti Hanni, X-ray scatter in projection radiography, Radiation Protection Dosimetry, 2023, ncad275, https://doi.org/10.1093/rpd/ncad275

In the originally published version of this manuscript, image panels (a), (b) and (c) of Figure 5 and those of Figure 7 were inadvertently transposed.

This has been corrected.

